# Environmental Carbon Monoxide Level Is Associated With the Level of High-Sensitivity C-Reactive Protein in Peritoneal Dialysis Patients

**DOI:** 10.1097/MD.0000000000000181

**Published:** 2014-12-05

**Authors:** Wen-Hung Huang, Tzung-Hai Yen, Ming-Jen Chan, Yi-Jiun Su

**Affiliations:** From the Department of Nephrology and Division of Clinical Toxicology (W-HH, T-HY); Department of Internal Medicine (M-JC, Y-JS), Chang Gung Memorial Hospital, Linkou; and Chang Gung University College of Medicine (W-HH, T-HY), Taoyuan, Taiwan.

## Abstract

Inflammation is highly prevalent among peritoneal dialysis (PD) patients. High-sensitivity C-reactive protein (hs-CRP) is the most widely used inflammatory marker in clinical medicine and is correlated with mortality in PD patients. Air pollution is associated with systemic inflammation. The aim of this cross-sectional study was to assess the role of air pollutants and other clinical variables on hs-CRP values in PD patients.

We recruited a total of 175 patients who had been undergoing continuous ambulatory PD or automated PD for at least 4 months and regularly followed up. Air pollution levels were recorded by a network of 27 monitoring stations near or in the patients’ living areas throughout Taiwan. The 12-month average concentrations of particulate matter (PM) with an aerodynamic diameter of <10 and <2.5 μm (PM_10_ and PM_2.5_), sulfur dioxide (SO_2_), nitrogen dioxide (NO_2_), carbon monoxide (CO), and ozone (O_3_) were included.

In stepwise linear regression, after adjustment for related factors, white blood cell count (*β*: 0.27, 95% confidence interval [CI] [0.71, 2.11]) and CO level (*β*: 0.17, 95% CI [2.5, 21.32]) were positively associated with hs-CRP and serum albumin levels (*β*: −0.25, 95% CI [−13.69, −3.96]) and normalized protein nitrogen appearance (*β*: −0.18, 95% CI [−17.7, −2.51]) was negatively associated with hs-CRP. However, serum indoxyl sulfate and *p*-cresyl sulfate levels were not significantly associated with hs-CRP (*P* > 0.05).

In PD patients, the environmental CO level was positively correlated with hs-CRP level.

## INTRODUCTION

The prevalence of inflammation among peritoneal dialysis (PD) patients is high. Inflammation is a powerful predictor of mortality and cardiovascular death in patients with end-stage renal disease (ESRD). The causes of inflammation in PD patients are complex and multifactorial, including factors related or unrelated to dialysis.^1^ The dialysis-related factors causing chronic inflammation in PD patients include peritoneal catheter use, glucose in the dialysate, peritonitis, exit-site infection, and endotoxins/cytokines from the dialysate; those factors unrelated to dialysis include loss of residual renal function,^2–5^ accumulation of uremic toxins, other comorbidities, malnutrition, or other infections.^1^ Chronic kidney disease (CKD) may act as a potent stimulus of inflammation.^6,7^ Protein-bound uremic toxins such as *p*-cresyl sulfate (PCS) and indoxyl sulfate (IS) are associated with atherosclerosis and endothelial dysfunction, and activation of atherosclerotic proinflammatory markers.^8–11^ C-reactive protein (CRP) is the most widely used inflammatory marker in clinical medicine. A change in residual kidney function has also been associated with an increase in CRP in PD patients.^12^ Several other studies reported a similar association between increased CRP level and increased mortality in both hemodialysis^13,14^ and PD patients.^3,15–17^ Interestingly, several studies have revealed that air pollution is associated with systemic inflammation.^18–20^ The 6 most common air pollutants are particulate matter (PM), lead (Pb), sulfur dioxide (SO_2_), nitrogen oxides (NO_*x*_), ozone (O_3_), and carbon monoxide (CO).^21^ Poor air quality has a profound and lasting effect on human health, particularly on the respiratory, cardiovascular,^22,23^ and central nervous systems.^24–26^ In a recent study, chronic CO exposure was associated with arterial wall thickness and an elevated level of high-sensitivity CRP (hs-CRP).^27^ To our knowledge, few studies have investigated air pollution as a dialysis-unrelated factor causing chronic inflammation in PD patients. The aim of this prospective cross-sectional study was to assess the role of air pollutants and other clinical variables on hs-CRP values in PD patients.

## MATERIALS AND METHODS

This study was approved by the ethical committee of the Chang Gung Memorial Hospital, Linkou, Taiwan, and performed in accordance with the principles of the Declaration of Helsinki. All the data were analyzed anonymously, and all patients’ records and information were anonymized and deidentified before analysis. Furthermore, all information was securely protected (by delinking identifying information from the main data set) and available to investigators only. Finally, all primary data were collected according to the Strengthening the Reporting of Observational Studies in Epidemiology guidelines.

### Study Population

Initially, we randomly recruited a total of 175 patients who had been undergoing continuous ambulatory PD (CAPD) or automated PD (APD) for at least 4 months and regularly followed up at a PD center in Chang Gung Memorial Hospital. All patients were randomly recruited between October 1 and November 30, 2009. Patients with a history of dialysis-related infection or other types of active infections within 3 months before study inclusion were excluded. PD supplies (CAPD and APD solutions) were obtained from Baxter Healthcare SA, Singapore. All patients gave their informed written consent. Age, gender, and clinical data were obtained from the patients’ medical records. All medical records during the study period, including medical history, laboratory data, and inclusion and exclusion factors, were reviewed by 2 nephrology specialists (W-HH and T-HY) and 1 general physician (M-JC).

### Sample Collection

Fasting blood, urine, and dialysate samples were collected on the same day between October 1 and November 30, 2009 during each patient's yearly routine examination, which included a peritoneal membrane function test. The plasma, dialysate, and urine concentrations of creatinine (Cr), serum albumin, and urea nitrogen were measured using routine laboratory methods. On the basis of the studies on the contributions of PCS and IS in the pathogenesis of vascular injury through endothelial dysfunction,^8,10^ proliferation of vascular smooth muscle cells,^9^ activation of atherosclerotic proinflammatory markers,^11^ and suppression of endothelial repair,^28^ we measured the serum concentrations of total formed IS and PCS for their correlation with the hs-CRP level. Protein nitrogen appearance was normalized to body weight (nPNA). Anuria was defined as 24-hour urine volume <50 cm^3^. Residual renal function was calculated as follows: (renal normalized urea nitrogen clearance + renal normalized Cr clearance)/2. Owing to the lack of any definite hs-CRP cutoff level indicating an inflammatory state in PD patients, inflammation was defined as an hs-CRP level of ≥10 mg/L, a level that has been correlated with increased mortality risk in PD patients.^17^ Levels of the air pollutants were also categorized into high and low according to the median value of each of the 6 air pollutants. The method for testing of IS and PCS was referenced from a previous study.^29^

### Air Quality Status and Analysis

To verify our inference that levels of air pollutants are correlated with hs-CRP values in PD patients, we analyzed the database and cited the report on the air quality status in Taiwan, using the data from the Taiwan Air Quality Monitoring Network operated by the Environmental Protection Administration.^30^ We recorded and analyzed the difference in air quality from the previous 12-month average exposure concentration of air pollutants according to the patients’ living areas. To the best of our knowledge, the appropriate averaging time for the air pollution effect on inflammation is unclear. Previous studies showed that the effect is spread overall for several days,^19,31^ months,^32^ or even years.^33^ From the above-cited studies, we considered the previous 12-month average exposure concentration of air pollutants for each subject's examination. The referenced items included previous 12-month average concentrations of PM with an aerodynamic diameter of <10 and <2.5 μm (PM_10_ and PM_2.5_), SO_2_, NO_2_, CO, and O_3_. Air pollution levels were recorded by a network of 27 monitoring stations near or in the patients’ living areas throughout Taiwan. The most northern living area is Hsichih District and the most southern area is Changhua City.

### Statistical Analysis

The Kolmogorov–Smirnov test was used to test if variables were to be normally distributed. A *P* value >0.05 was required to assume a normal distribution. Data are expressed in terms of median and interquartile range in nonnormal distribution variables and as mean ± standard deviation in normal distribution variables. Comparisons between groups were performed using the Mann–Whitney test and Student *t* test. χ^2^ or Fisher exact tests were used to analyze the correlation between categorical variables. To calculate the relative correlation of the hs-CRP value, standardized coefficients (*β*) and 95% CIs were obtained using linear regression models. Univariate and stepwise linear regression analyses were used. The following factors were investigated: PM_10_, SO_2_, NO_2_, CO, O_3_, PM_2.5_, age, PD duration, serum Cr level, white blood cell count (WBC), nPNA, serum albumin, serum total IS, serum total PCS, residual renal function test, intact parathyroid hormone level (iPTH), blood aluminum (Al) level, diabetes mellitus (DM), coronary artery disease (CAD), and hypertension. All the nominal variables in linear regression were transformed into dummy coding. Missing data was approached with listwise deletion. All statistical analyses were performed using the Statistical Package for the Social Sciences, version 12.0 for Windows (SPSS Inc, Chicago, IL). A *P* value of <0.05 was considered statistically significant.

## RESULTS

### Patient Characteristics

A total of 175 patients from a single PD center were enrolled in this study. Fourteen patients received APD and 161 patients received CAPD. Table 1 lists the characteristics of the study subjects (mean age, 50 years). Of all patients, 125 were women and 78 patients showed anuria. Furthermore, the daily exchange volume was 9.85 L. The median hs-CRP level was 2.8 mg/L (range, 1.2–7.6 mg/L). Fifteen patients (8.5%) were habitual users of tobacco. The median concentration of PM_10_ was 49.1 μg/m^3^; SO_2_, 5.2 ppb; NO_2_, 20.1 ppb; CO, 0.53 ppm; O_3_, 28.7 ppb; and PM_2.5_, 29.6 μg/m^3^. The causes of ESRD were diabetic nephropathy (n = 21), polycystic kidney disease (n = 1), glomerular disease (n = 79), malignant hypertension (n = 15), obstructive nephropathy (n = 3), lupus nephritis (n = 4), gouty nephropathy (n = 2), tubulointerstitial disease (n = 2), and unknown factors (n = 48).

**TABLE 1:**
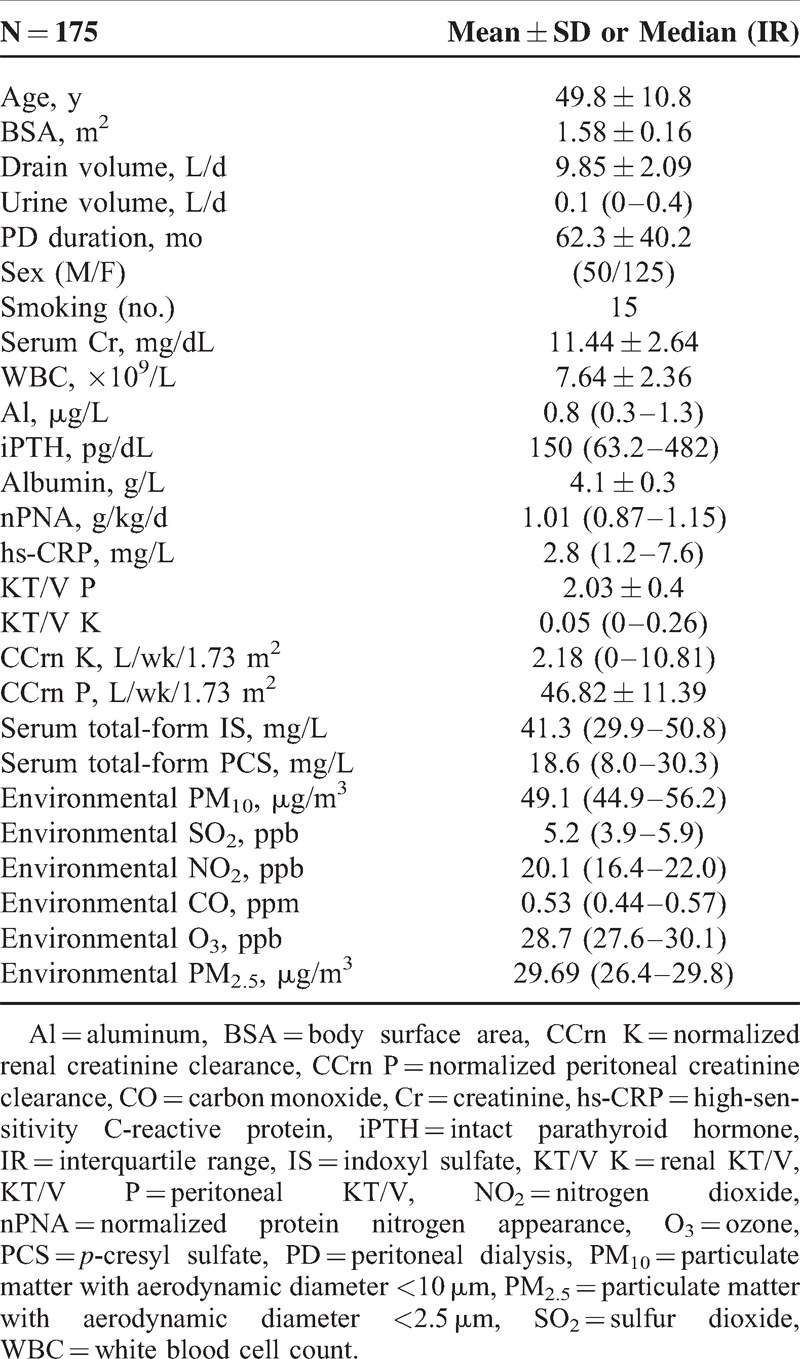
Characteristics of the 175 Peritoneal Dialysis Patients

### Factors Associated With hs-CRP Level in Patients With PD

**FIGURE 1:**
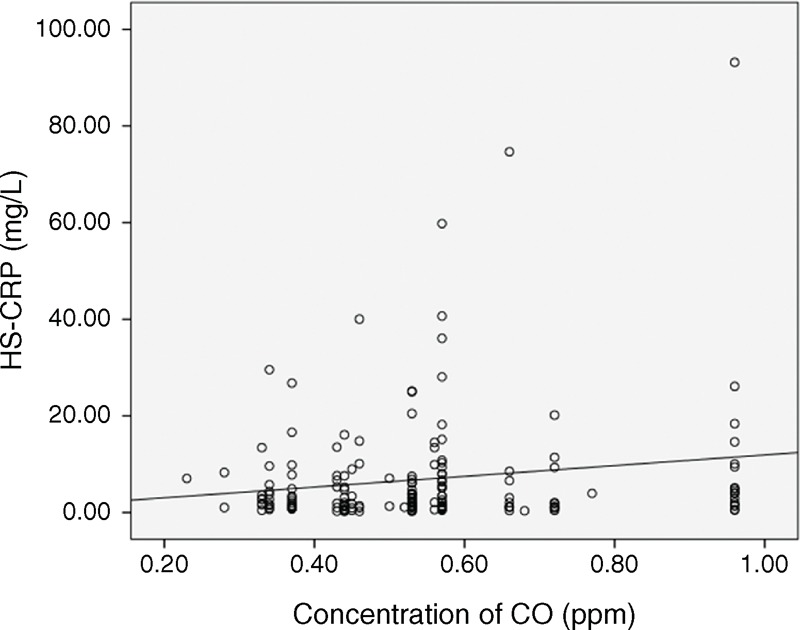
Relationship between airborne CO and hs-CRP (*r* = 0.16, *P* = 0.03). CO = carbon monoxide, hs-CRP = high-sensitivity C-reactive protein.

The correlation between CO and hs-CRP was revealed in Figure 1 (*r* = 0.16, *P* = 0.03). To further investigate the correlation between inflammation (hs-CRP ≥10 mg/L) and the 6 air pollutants, we categorized the levels into high and low levels according to each pollutant's median value and analyzed the results with the linear trend for ordinal variables analysis. The median value of PM_10_ was 49.1 μg/m^3^; SO_2_, 5.2 ppb; NO_2_, 20.1 ppb; CO, 0.53 ppm; O_3_, 28.7 ppb; and PM_2.5_, 29.6 μg/m^3^. Table 2 shows that among the 6 air pollutants, a high level of CO was significantly (*P* = 0.04) associated with a high level of hs-CRP (≥10 mg/L); however, all the other pollutants did not show a significant association (*P* > 0.05). In this study, 75 patients lived in the area of higher level of CO and other 100 patients lived with contrary condition. Thirty patients had inflammation status. Except for gender, no related clinical variables were different between these 2 groups (Table 3). To further clarify the factors associated with hs-CRP level in our study patients, we used univariate and multivariate linear regression “stepwise” methods, for analyses. PM_10_, SO_2_, NO_2_, CO, O_3_, PM_2.5_, age, PD duration, serum Cr level, WBC, nPNA, serum albumin level, serum total IS, serum total PCS, residual renal function test, iPTH, blood Al level, DM, CAD, and hypertension were investigated as clinical variables. Table 4 revealed that in univariate linear regression, the environmental CO level (*β*: 0.16, 95% CI [0.91, 21.27]), blood Al level (*β*: 0.16, 95% CI [0.17, 3.91]), and blood WBC (*β*: 0.24, 95% CI [0.51, 1.98]) were positively associated with hs-CRP and serum albumin levels (*β*: −0.22, 95% CI [−12.95, −2.78]), and log nPNA (*β*: −0.24, 95% CI [−50.47, −12.75]) was negatively associated with hs-CRP level. Except for CO, the other air pollutants were not associated with hs-CRP level (*P* > 0.05). Furthermore, in the “stepwise” model of linear regression, after adjustment for related factors, blood WBC (*β*: 0.28, 95% CI [0.73, 2.12]) and CO level (*β*: 0.17, 95% CI [2.54, 21.27]) were positively associated with hs-CRP and serum albumin levels (*β*: −0.25, 95% CI [−13.51, −3.93]), and log nPNA (*β*: −0.18, 95% CI [−17.25, −2.33]) was negatively associated with hs-CRP level. However, serum IS and PCS levels were not significantly associated with hs-CRP level (*P* > 0.05).

**TABLE 2:**
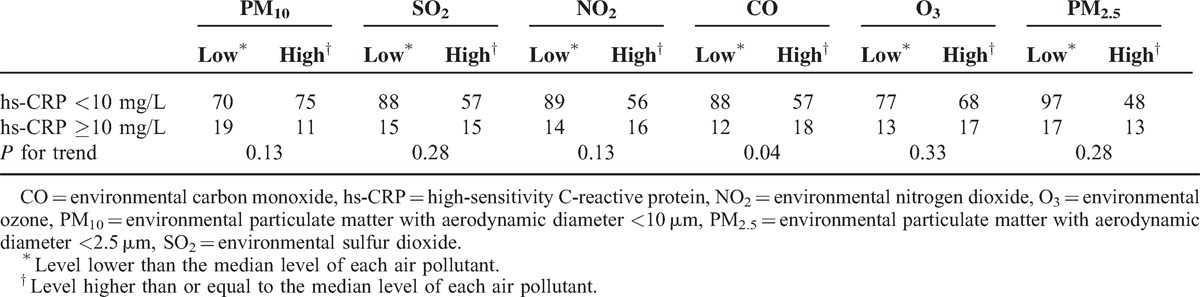
Linear Trend for Ordinal Variables Analysis of Air Pollutants and hs-CRP

**TABLE 3:**
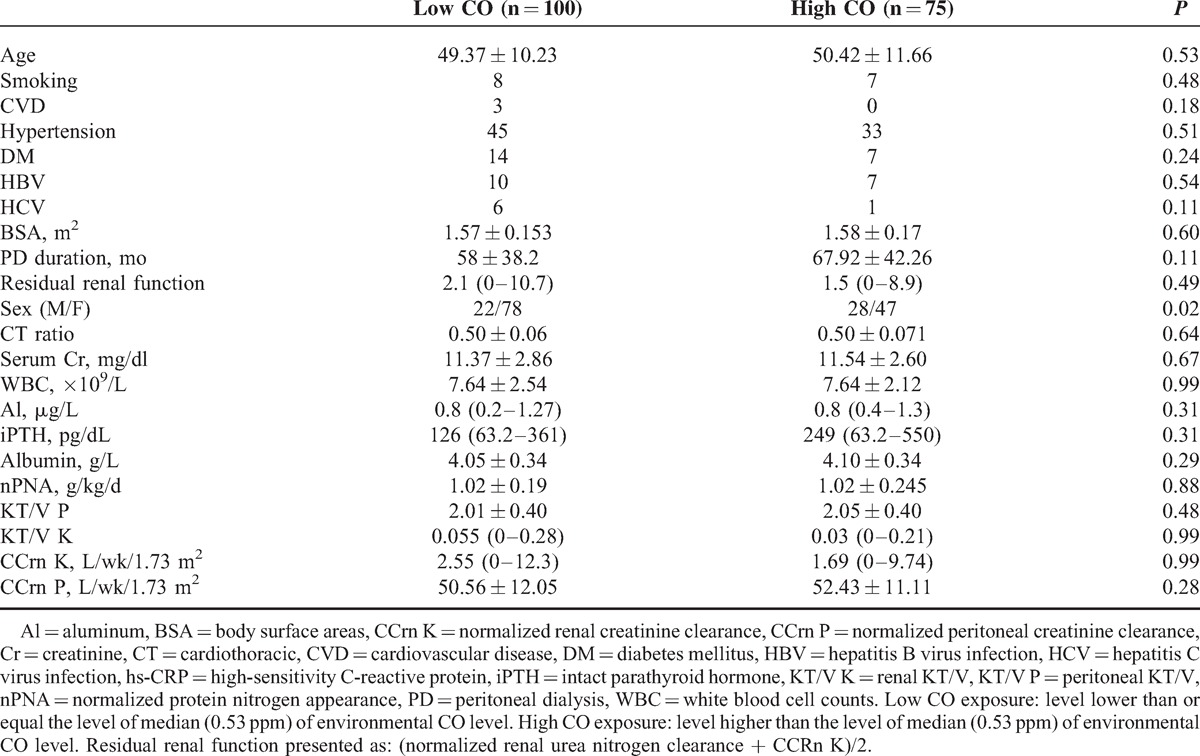
Comparison of Patients With Low and High-Environmental CO Exposure

**TABLE 4:**
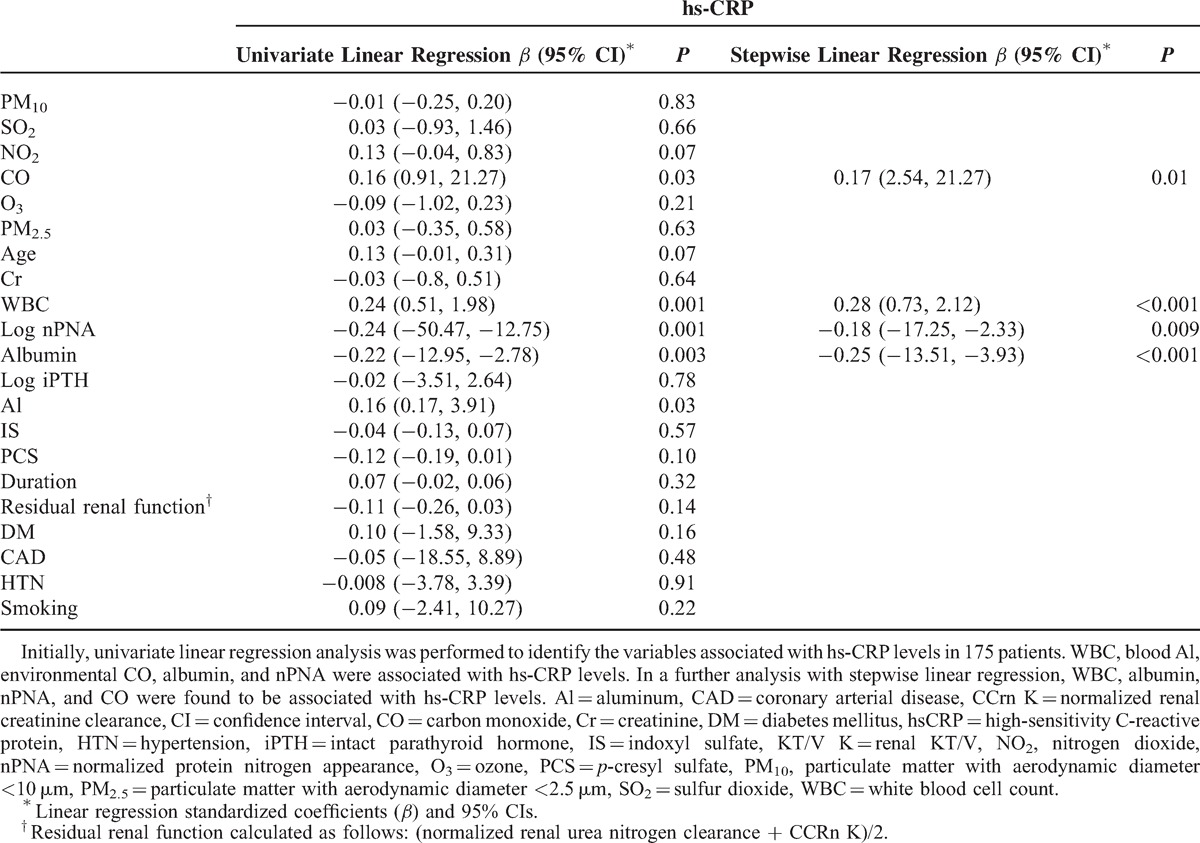
Linear Regression Analysis Between Clinical Variables and hs-CRP Level (N = 175)

## DISCUSSION

In this study, we have shown that after adjustment for related risk factors, the level of the air pollutant CO was significantly positively associated with the level of the inflammation marker hs-CRP in PD patients.

It is well known that cardiovascular disease (CVD) is the leading cause of death in patients with ESRD, and chronic inflammation is a risk factor correlated with CVD. Inflammation is highly prevalent in the PD population. When estimated by CRP level with different cutoff values and assay sensitivities, the prevalence of inflammation varied between 12% and 65%.^34^ The causes of inflammation in PD patients are multifactorial,^1,35^ primarily systemic infection, glucose in the dialysate, peritonitis, malnutrition, residual renal function, and accumulation uremic toxins.^1,35^ Bergstrom^36^ showed an important association between increased CRP level and increased mortality in hemodialysis patients. A number of other studies reported a similar association between increased CRP level and increased mortality in both hemodialysis^13,14^ and PD patients.^3,15,16^ Herzig et al^15^ showed in a study of 50 PD patients that an increased CRP level was associated with an increased risk of acute myocardial infarction. Wang et al^3^ also pointed out that an increased CRP level predicts mortality and cardiovascular death independent of other clinical, cardiovascular, dialysis, nutritional, and biochemical parameters. Iseki et al^17^ reported that in a 5-year follow-up study, a CRP level of 10 mg/L or higher was associated with a >3-fold increased mortality in PD patients. An increased CRP level has also been shown to predict cardiovascular events in PD patients independent of other traditional and nontraditional risk factors.^37^

Interestingly, several studies have revealed that air pollution is associated with systemic inflammation.^18–20^ The 6 most common air pollutants are PM, Pb, SO_2_, NO_*x*_, O_3_, and CO.^21^ The effects of poor air quality on human health are profound and lasting, and principally affect the respiratory and cardiovascular systems.^22,23^ In addition, air pollution has been associated with increased incidence of and mortality from CAD.^38^ In a large population and long follow-up study of 6 US cities (Harvard Six Cities study),^22^ a significant association was noted between air pollution and mortality after adjustment for smoking, particularly in cities where pollutant levels were highest. An extended follow-up of the Harvard Six Cities study showed that cardiovascular and lung cancer mortality rates were each positively associated with fine particulate air pollution (PM_2.5_).^39^

To our knowledge, reports on the association between inflammation and air pollution in PD patients are limited. In this study, we found that the CO level is positively associated with the level of hs-CRP after adjustment for related risk factors. CO is a colorless, odorless, and tasteless gas that is slightly lighter than air. It occurs in various natural and artificial environments. However, with increasing industrialization and urbanization, CO is chiefly emitted from the exhaust of internal combustion engines and industrial production, as well as from incomplete combustion of various other fuels. CO is toxic to humans and animals at higher concentrations. Acute CO poisoning is known to occur worldwide, and its major complications involve the central nervous and cardiovascular systems. However, our knowledge about the effects of chronic CO poisoning is limited, especially in PD patients. The results of chronic CO exposure in human and animal studies are conflicting. Many animal studies^40–42^ have shown that short-term and low-level CO exposure may protect against inflammatory and reactive oxygen-inducing stimuli. In other studies, intermittent CO exposure caused damage to the arterial wall and resulted in atherosclerosis.^43–45^ Epidemiological studies have shown that high levels of environmental CO were correlated with CVD and mortality.^46,47^ In a recent study, Davutoglu et al^27^ showed that hs-CRP levels and carotid intima-media thickness are increased in subjects with chronic CO exposure. The mechanism by which chronic CO exposure increases hs-CRP level is not clear. However, in the study by Davutoglu et al, one limitation is that environmental CO level and other air pollutants were not measured. From the above-cited studies, we could infer that short-term and low-level CO exposure may have anti-inflammatory effects in animals. However, chronic CO exposure may damage the arterial wall and result in atherosclerosis. In our study, we found that in PD patients, the environmental CO level was positively correlated with hs-CRP level. This result agrees with the findings of the studies cited above.

## CONCLUSION

In conclusion, this cross-sectional study shows that in patients with PD, the environmental CO level is associated with the level of hs-CRP. Further studies are required to clarify the role of environmental air pollutants in inflammation and related comorbidities in PD patients.

### Limitations

This study has several limitations. Among them is the cross-sectional nature of the observation and that the study group is predominantly represented by women. In our PD center, the patients themselves choose between PD and HD. Considering the working environment and home-care conditions, most women chose PD. On the contrary, according to the limitations in technology and information on indoor air quality, we used the previous 1-year average air quality published by the Environmental Protection Administration Executive Yuan, Taiwan to represent the air quality of the patients’ living areas.

## References

[1] Wang AY Consequences of chronic inflammation in peritoneal dialysis. *Semin Nephrol* 2011; 31:159–171. 21439430 10.1016/j.semnephrol.2011.01.005

[2] Wang AY Lam CW Chan IH Long-term mortality and cardiovascular risk stratification of peritoneal dialysis patients using a combination of inflammation and calcification markers. *Nephrol Dial Transplant* 2009; 24:3826–3833. 19574337 10.1093/ndt/gfp325

[3] Wang AY Woo J Lam CW Is a single time point C-reactive protein predictive of outcome in peritoneal dialysis patients? *J Am Soc Nephrol* 2003; 14:1871–1879. 12819248 10.1097/01.asn.0000070071.57901.b3

[4] Wang AY Lam CW Chan IH Prognostic value of plasma myeloperoxidase in ESRD patients. *Am J Kidney Dis* 2010; 56:937–946. 20638167 10.1053/j.ajkd.2010.05.008

[5] Wang AY Lam CW Wang M Circulating soluble vascular cell adhesion molecule 1: relationships with residual renal function, cardiac hypertrophy, and outcome of peritoneal dialysis patients. *Am J Kidney Dis* 2005; 45:715–729. 15806475 10.1053/j.ajkd.2004.12.012

[6] Glorieux G Cohen G Jankowski J Platelet/leukocyte activation, inflammation, and uremia. *Semin Dial* 2009; 22:423–427. 19708994 10.1111/j.1525-139X.2009.00593.x

[7] Herbelin A Urena P Nguyen AT Elevated circulating levels of interleukin-6 in patients with chronic renal failure. *Kidney Int* 1991; 39:954–960. 2067212 10.1038/ki.1991.120

[8] Yu M Kim YJ Kang DH Indoxyl sulfate-induced endothelial dysfunction in patients with chronic kidney disease via an induction of oxidative stress. *Clin J Am Soc Nephrol* 2011; 6:30–39. 20876676 10.2215/CJN.05340610 PMC3022246

[9] Shimizu H Hirose Y Nishijima F ROS and PDGF-beta [corrected] receptors are critically involved in indoxyl sulfate actions that promote vascular smooth muscle cell proliferation and migration. *Am J Physiol Cell Physiol* 2009; 297:C389–396. 19494236 10.1152/ajpcell.00206.2009

[10] Meijers BK Van Kerckhoven S Verbeke K The uremic retention solute p-cresyl sulfate and markers of endothelial damage. *Am J Kidney Dis* 2009; 54:891–901. 19615803 10.1053/j.ajkd.2009.04.022

[11] Dou L Jourde-Chiche N Faure V The uremic solute indoxyl sulfate induces oxidative stress in endothelial cells. *J Thromb Haemost* 2007; 5:1302–1308. 17403109 10.1111/j.1538-7836.2007.02540.x

[12] Chung SH Heimburger O Stenvinkel P Association between inflammation and changes in residual renal function and peritoneal transport rate during the first year of dialysis. *Nephrol Dial Transplant* 2001; 16:2240–2245. 11682675 10.1093/ndt/16.11.2240

[13] Zimmermann J Herrlinger S Pruy A Inflammation enhances cardiovascular risk and mortality in hemodialysis patients. *Kidney Int* 1999; 55:648–658. 9987089 10.1046/j.1523-1755.1999.00273.x

[14] Wanner C Metzger T C-reactive protein a marker for all-cause and cardiovascular mortality in haemodialysis patients. *Nephrol Dial Transplant* 2002; 17 Suppl 8:29–32. 12147774 10.1093/ndt/17.suppl_8.29

[15] Herzig KA Purdie DM Chang W Is C-reactive protein a useful predictor of outcome in peritoneal dialysis patients? *J Am Soc Nephrol* 2001; 12:814–821. 11274243 10.1681/ASN.V124814

[16] Noh H Lee SW Kang SW Serum C-reactive protein: a predictor of mortality in continuous ambulatory peritoneal dialysis patients. *Perit Dial Int* 1998; 18:387–394. 10505560

[17] Iseki K Tozawa M Yoshi S Fukiyama K Serum C-reactive protein (CRP) and risk of death in chronic dialysis patients. *Nephrol Dial Transplant* 1999; 14:1956–1960. 10462277 10.1093/ndt/14.8.1956

[18] Rich DQ Kipen HM Huang W Association between changes in air pollution levels during the Beijing Olympics and biomarkers of inflammation and thrombosis in healthy young adults. *JAMA* 2012; 307:2068–2078. 22665106 10.1001/jama.2012.3488 PMC4049319

[19] Bind MA Baccarelli A Zanobetti A Air pollution and markers of coagulation, inflammation, and endothelial function: associations and epigene-environment interactions in an elderly cohort. *Epidemiology* 2012; 23:332–340. 22237295 10.1097/EDE.0b013e31824523f0 PMC3285258

[20] Alexeeff SE Coull BA Gryparis A Medium-term exposure to traffic-related air pollution and markers of inflammation and endothelial function. *Environ Health Perspect* 2011; 119:481–486. 21349799 10.1289/ehp.1002560 PMC3080929

[21] Samet JM The Clean Air Act and health—a clearer view from 2011. *N Engl J Med* 2011; 365:198–201. 21732828 10.1056/NEJMp1103332

[22] Dockery DW Pope CA 3rd Xu X An association between air pollution and mortality in six U.S. cities. *N Engl J Med* 1993; 329:1753–1759. 8179653 10.1056/NEJM199312093292401

[23] Dockery DW Stone PH Cardiovascular risks from fine particulate air pollution. *N Engl J Med* 2007; 356:511–513. 17267912 10.1056/NEJMe068274

[24] Mateen FJ Brook RD Air pollution as an emerging global risk factor for stroke. *JAMA* 2011; 305:1240–1241. 21427378 10.1001/jama.2011.352

[25] Tsai SS Goggins WB Chiu HF Yang CY Evidence for an association between air pollution and daily stroke admissions in Kaohsiung, Taiwan. *Stroke* 2003; 34:2612–2616. 14551399 10.1161/01.STR.0000095564.33543.64

[26] Miller KA Siscovick DS Sheppard L Long-term exposure to air pollution and incidence of cardiovascular events in women. *N Engl J Med* 2007; 356:447–458. 17267905 10.1056/NEJMoa054409

[27] Davutoglu V Zengin S Sari I Chronic carbon monoxide exposure is associated with the increases in carotid intima-media thickness and C-reactive protein level. *Tohoku J Exp Med* 2009; 219:201–206. 19851048 10.1620/tjem.219.201

[28] Dou L Bertrand E Cerini C The uremic solutes p-cresol and indoxyl sulfate inhibit endothelial proliferation and wound repair. *Kidney Int* 2004; 65:442–451. 14717914 10.1111/j.1523-1755.2004.00399.x

[29] Huang WH Hung CC Yang CW Huang JY High correlation between clearance of renal protein-bound uremic toxins (indoxyl sulfate and p-cresyl sulfate) and renal water-soluble toxins in peritoneal dialysis patients. *Ther Apher Dial* 2012; 16:361–367. 22817125 10.1111/j.1744-9987.2012.01068.x

[30] Taiwan Air Quality Monitoring Network (TAQMN) operated by the Environmental Protection Administration (EPA). Air quality monitoring data of 2008 and 2009. http://taqm.epa.gov.tw/taqm/zh-tw/default.aspx Accessed January 6, 2014

[31] Peters A Frohlich M Doring A Particulate air pollution is associated with an acute phase response in men; results from the MONICA-Augsburg Study. *Eur Heart J* 2001; 22:1198–1204. 11440492 10.1053/euhj.2000.2483

[32] Zanobetti A Schwartz J Samoli E The temporal pattern of respiratory and heart disease mortality in response to air pollution. *Environ Health Perspect* 2003; 111:1188–1193. 12842772 10.1289/ehp.5712 PMC1241573

[33] Panasevich S Leander K Rosenlund M Associations of long- and short-term air pollution exposure with markers of inflammation and coagulation in a population sample. *Occup Environ Med* 2009; 66:747–753. 19687019 10.1136/oem.2008.043471

[34] Wang AY Prognostic value of C-reactive protein for heart disease in dialysis patients. *Curr Opin Investig Drugs* 2005; 6:879–886. 16187687

[35] Lai KN Leung JC Inflammation in peritoneal dialysis. *Nephron Clin Pract* 2010; 116:c11–c18. 20460934 10.1159/000314544

[36] Bergstrom J Inflammation, malnutrition, cardiovascular disease and mortality in end-stage renal disease. *Pol Arch Med Wewn* 2000; 104:641–643. 11392151

[37] Ducloux D Bresson-Vautrin C Kribs M C-reactive protein and cardiovascular disease in peritoneal dialysis patients. *Kidney Int* 2002; 62:1417–1422. 12234314 10.1111/j.1523-1755.2002.kid562.x

[38] Gan WQ Davies HW Koehoorn M Brauer M Association of long-term exposure to community noise and traffic-related air pollution with coronary heart disease mortality. *Am J Epidemiol* 2012; 175:898–906. 22491084 10.1093/aje/kwr424

[39] Laden F Schwartz J Speizer FE Dockery DW Reduction in fine particulate air pollution and mortality: extended follow-up of the Harvard Six Cities study. *Am J Respir Crit Care Med* 2006; 173:667–672. 16424447 10.1164/rccm.200503-443OC PMC2662950

[40] Mitchell LA Channell MM Royer CM Evaluation of inhaled carbon monoxide as an anti-inflammatory therapy in a nonhuman primate model of lung inflammation. *Am J Physiol Lung Cell Mol Physiol* 2010; 299:L891–897. 20729385 10.1152/ajplung.00366.2009

[41] Wunder C Brock RW Frantz S Carbon monoxide, but not endothelin-1, plays a major role for the hepatic microcirculation in a murine model of early systemic inflammation. *Crit Care Med* 2005; 33:2323–2331. 16215388 10.1097/01.ccm.0000182794.42733.71

[42] Sass G Soares MC Yamashita K Heme oxygenase-1 and its reaction product, carbon monoxide, prevent inflammation-related apoptotic liver damage in mice. *Hepatology* 2003; 38:909–918. 14512878 10.1053/jhep.2003.50386

[43] Astrup P Kjeldsen K Wanstrup J Enhancing influence of carbon monoxide on the development of atheromatosis in cholesterol-fed rabbits. *J Atheroscler Res* 1967; 7:343–354. 6036104 10.1016/s0368-1319(67)80061-9

[44] Astrup P Kjeldsen K Wanstrup J Effects of carbon monoxide exposure on the arterial walls. *Ann N Y Acad Sci* 1970; 174:294–300. 5289606 10.1111/j.1749-6632.1970.tb49796.x

[45] Smith CJ Steichen TJ The atherogenic potential of carbon monoxide. *Atherosclerosis* 1993; 99:137–149. 8503943 10.1016/0021-9150(93)90017-o

[46] Stern FB Halperin WE Hornung RW Heart disease mortality among bridge and tunnel officers exposed to carbon monoxide. *Am J Epidemiol* 1988; 128:1276–1288. 2461656 10.1093/oxfordjournals.aje.a115081

[47] Kleinman MT Davidson DM Vandagriff RB Effects of short-term exposure to carbon monoxide in subjects with coronary artery disease. *Arch Environ Health* 1989; 44:361–369. 2692521 10.1080/00039896.1989.9935908

